# How Does SARS-CoV-2 Affect Our Eyes—What Have We Learnt So Far about the Ophthalmic Manifestations of COVID-19?

**DOI:** 10.3390/jcm11123379

**Published:** 2022-06-13

**Authors:** Jacek Baj, Alicja Forma, Barbara Teresińska, Magdalena Tyczyńska, Julita Zembala, Jacek Januszewski, Jolanta Flieger, Grzegorz Buszewicz, Grzegorz Teresiński

**Affiliations:** 1 Chair and Department of Anatomy, Medical University of Lublin, 20-090 Lublin, Poland; m.tyczynska@onet.pl (M.T.); jacek.januszewski000@gmail.com (J.J.); 2 Chair and Department of Forensic Medicine, Medical University of Lublin, 20-090 Lublin, Poland; b.teresinska2@gmail.com (B.T.); g.buszewicz@umlub.pl (G.B.); grzegorz.teresinski@umlub.pl (G.T.); 3 Department of Ophthalmology, Medical University of Warsaw, 02-091 Warsaw, Poland; zembalajulita@gmail.com; 4 Department of Analytical Chemistry, Medical University of Lublin, 20-093 Lublin, Poland; j.flieger@umlub.pl

**Keywords:** ophthalmic manifestations, SARS-CoV-2, atypical symptom, COVID-19, ophthalmology

## Abstract

The severe acute respiratory syndrome coronavirus-2 (SARS-CoV-2) infection has become a worldwide threat resulting in a pandemic in 2020. SARS-CoV-2 infection manifests itself as coronavirus disease 2019 (COVID-19) that is evidenced in a vast number of either specific or nonspecific symptoms. Except for typical (but nonspecific) symptoms such as fever, dry cough, or muscle weakness, the infected patients might also present atypical symptoms including neurological, dermatological, or ophthalmic manifestations. This paper summarizes the current state of knowledge regarding the onset, progression, and types of ophthalmic symptoms induced by SARS-CoV-2 infection recognized amongst the infected patients.

## 1. Introduction

Severe acute respiratory syndrome, coronavirus 2 (SARS-CoV-2), which can cause coronavirus disease 2019 (COVID-19), was first observed in the city of Wuhan, China in late 2019 [1]. The infection induced by the virus can spread rapidly and may lead to serious systemic complications, especially those associated with the respiratory system [1,2]. The disease was announced as a pandemic in March 2020 with an infection rate of about 291,000,000 between December 2019 and January 2022, causing nearly 5,500,000 deaths [2,3]. SARS-CoV-2 is a single, positive-stranded RNA beta coronavirus that has transmitted and spread all over the world since January 2020 [2,4]. The transmission (mainly by aerosol and droplets) is related to the angiotensin-converting enzyme 2 (ACE2) receptor located in the membrane of the lungs, heart, kidneys, or ocular cells to which the coronavirus attaches [4]. COVID-19 manifests itself as flu-like condition but can expose many other symptoms, frequently of an unusual nature [4].

SARS-CoV-2 impacts different systems of the human body. Some patients exhibit nonspecific symptoms, such as headache, nausea, vomiting, dizziness, and confusion, while some present with more specific ones such as: seizures and cerebrovascular disorders [5,6]. COVID-19 can also lead to some serious cardiovascular complications [7]. Furthermore, the frequency of acute kidney injury (AKI) in patients with COVID-19 is quite common; besides that, SARS-CoV-2 presented a tropism in female and male reproductive organs [8,9]. What is more, some cutaneous manifestations of COVID-19, which include maculopapular, chilblain-like, urticarial, vesicular, livedoid, and petechial lesions, are observed (Table 1).

Pediatric patients with SARS-CoV-2 usually have a higher rate of mild infection than adults and present fewer complications. Over one-third of pediatric patients present normal chest computed tomography (CT) scans. The most common radiological findings regardless of patients’ age include ground glass opacities and the presence of consolidations or pneumonic infiltrates.

Most infected patients prove to have a higher rate of the following biomarkers—C-reactive protein, serum amyloid A, interleukin-6, lactate dehydrogenase, neutrophil-to-lymphocyte ratio, D-dimer, cardiac troponin, renal biomarkers, lymphocytes, and platelet Mount [10]. Concerning the accuracy of SARS-CoV-2 diagnostic tests, RT-PCR remains the gold standard for COVID-19 diagnosis. The combination of IgM and IgG antibodies also demonstrated promising results as for sensitivity and specificity [11].

## 2. Pathophysiology and Transmission of SARS-CoV-2 Infection

SARS-CoV-2 is a 45726 single-stranded positive-sense RNA virus classified as betacoronaviridae. It can spread in many ways in the form of aerosols, droplets, or conjunctival transmission [1,12]. Following the entry, mainly due to the ACE-2 receptor, the virion binds to the host cells and enters the cells through endocytosis or membrane fusion [11]. ACE-2 receptors are found in almost every organ, such as the lungs, heart, kidneys, or gastrointestinal system. This could partially explain some atypical symptoms and dangerous complications of SARS-CoV-2 infection [1,13]. As presented earlier, following entry, the virus releases its RNA in the epithelial cells (ECs) [1]. In infected Ecs, inflammation; abnormal cytokine release (VEGF, MCP-1, and IL-8); and tissue damage are consecutively observed [1]. All these disorders might eventually lead to multiorgan failure or acute respiratory distress syndrome (ARDS) [1].

Transmission is possible owing to droplets and other body fluids, air, and fomite. There is also a possibility of conjunctival transmission; aerosols < 5 μm in diameter can cause airborne transmission [14]. Infected tears or respiratory droplets may come into contact with the conjunctiva and lead to SARS-CoV-2 infection [15]. In the following review, we aimed to summarize and briefly present the current state of our knowledge regarding examples of possible ophthalmic manifestations of SARS-CoV-2 infection.

## 3. Aim of the Review and Search Strategy

The objective of this paper was to conduct a scoping review regarding the possible ophthalmic manifestations associated with the SARS-CoV-2 infection. The review aims to present the ophthalmic diseases that might occur due to infection, as well as to evaluate the progression of the symptoms. The review of the literature was performed with the use of three databases—PubMed, Scopus, and Web of Science—until 30th March 2022. The search string was as follows—the first identification of the articles was performed using the following keywords—(COVID-19 OR SARS-CoV-2) AND (ophthalmic). After the first identification, amongst 3292 articles, the ones associated with the organ of vision and possible ophthalmic manifestations that might occur due to the SARS-CoV-2 infection were chosen. Thus, the second identification of the articles included the following search string—(COVID-19 OR SARS-CoV-2) AND (conjunctivitis OR episcleritis OR keratoconjunctivitis OR central retinal vein occlusion OR vitritis OR Kawasaki disease OR ophthalmoparesis OR acute retinal necrosis OR oculomotor nerve palsy OR ptosis OR papillophlebitis OR optic neuritis OR Adie’s tonic pupil OR keratitis OR cerebrovascular accident OR dacryoadenitis OR glaucoma OR Horner’s syndrome OR retro-orbital pain OR Miller Fisher syndrome OR orbital ellulitis OR orbital sinusitis OR conjunctival chemosis OR xerophthalmia OR mucormycosis). Amongst 5551 articles, 115 were found to be associated with the topic of this study, and they were included in the qualitative synthesis. The literature search exclusively covered the human studies. There were no restrictions regarding the year of the publication; however, the authors chose only the articles in English.

## 4. Anterior Segment of the Eye

### 4.1. Follicular Conjunctivitis

Conjunctivitis has, so far, been the main ophthalmic manifestation documented in COVID-19 patients in the literature. The first case of conjunctivitis associated with SARS-CoV-2 infection was described as unilateral ocular redness preceding pneumonia [16]. A recent meta-analysis pointed out that, out of 8219 patients with confirmed SARS-CoV-2 infection, only 11.03% reported ocular manifestations [17]. The most common complaint was a foreign body sensation, which was reported in 16% of the ocular cases, followed by redness (13.3%) and tearing (12.8%). Most ophthalmic conditions were associated with conjunctivitis (88.8%), while 4.4% of cases were diagnosed with either keratitis or keratoconjunctivitis [18]. Another meta-analysis suggested that there is a relation between conjunctivitis and the worsening of the manifestations of COVID-19 [19]. It was also reported that the prevalence of conjunctivitis in COVID-19 patients is estimated to be relatively low (5.5% (42/735 patients)), with the PCR of lacrimal exudate being scarcely sensitive (0.6%) but very specific (100%) compared to a nasopharyngeal swab. In one of the largest published series, only 9 (0.8%) out of 1099 patients developed ocular congestion [20]. The study of 534 patients showed that conjunctivitis was only observed in 25 (4.68%) patients [21]. A cohort of 30 patients with the SARS-CoV-2 infection showed the presence of the virus only in one eye of the patient without any complications [22]. A retrospective series of 67 patients with COVID-19 reported no patients with conjunctivitis [23]; however, these patients presented positive conjunctival exudate. Likewise, in another study involving 17 COVID-19 patients, SARS-CoV-2 was not found in the conjunctiva in repeated shots, although one patient developed unilateral eye redness [24]. The other study, 102 patients with COVID-19 showed that only 2 patients presented conjunctivitis, and again, only one of them had SARS-CoV-2 detected within the ocular surface [25]. In a series of 38 COVID-19 patients, up to 31% of ocular complications were described (12 patients with ocular congestion) [26]. In this study, the positive correlation between the severity of the systemic infection and ocular manifestation was highlighted by the authors. It was also concluded that conjunctivitis could constitute the risk factor for severe systemic infection when it appears in the intermediate phase of infection. It was reported that conjunctival manifestations might appear even before the onset of respiratory symptoms [27].

A large series of case studies showed that 8.66% (11/127) of patients diagnosed with COVID-19 had conjunctivitis [28]. The conjunctival congestion was associated with the presence of respiratory tract symptoms. In this study, the authors reported that hand–eye contact was not clinically significant. Another large study reported that hand–eye contact was independently correlated to the presence of conjunctival congestion [21]. So far, five cases of acute follicular conjunctivitis that were the first and sole manifestation of COVID-19 have been reported [29].

Until now, the majority of published cases have stated that the manifestations of conjunctivitis associated with the SARS-CoV-2 infection are mild, of bilateral character, follicular type, and the majority present without corneal symptoms; additionally, the symptoms can coincide with another conjunctivitis of another viral origin, such as adenovirus, for instance. It was found that ocular manifestations are more common in the middle phase of COVID-19 based on the findings of bilateral acute follicular conjunctivitis in the patient on the 13th day of the illness [21].

### 4.2. Viral Keratoconjunctivitis

To date, three cases in the literature have reported keratoconjunctivitis in COVID-19 patients. Keratoconjunctivitis, as the initial presentation of COVID-19 disease, was confirmed in the case study of a 29-year-old woman who presented with unilateral redness, photophobia, and watery discharge and was primarily diagnosed with herpetic keratoconjunctivitis and treated with oral valacyclovir and moxifloxacin in drops without any improvement [30]. The PCR test was positive. The CT values of the simultaneous RT-PCR of nasopharynx and conjunctival swabs were for 23 and 37 cycles, respectively.

Alnajjar et al., reported a case of a 24-h lag between the presentation of the keratoconjunctivitis symptoms and the onset of respiratory manifestations [18]. There was a four-day gap between taking a nasopharynx swab and an eye swab, with PCR results of positive (CT value 26) and negative (i.e., CT > 40 cycle) values, respectively. In the third reported case of the relapse of keratoconjunctivitis, the RT-PCR test of a conjunctival sac swab was negative [31]. The inflammatory cytokines were high (especially Il-6) in the eye secretions, which could suggest that the disease was caused by a local cytokine storm rather than a direct invasion of the virus.

### 4.3. Hemorrhagic and Pseudomembranous Conjunctivitis

The first case of pseudomembranous and hemorrhagic conjunctivitis related to SARS-CoV-2-induced pneumonia in a patient in an intensive care unit (ICU) was described in France. Hemorrhagic and pseudomembranous conjunctivitis started late in the natural evolution of COVID-19, developing 19 days after the onset of systemic symptoms [32].

### 4.4. Keratitis

Infectious keratitis remains a major global cause of visual impairment and blindness. The reactivation of herpesviruses is widespread among critically ill patients, including those with severe COVID-19. Majtanova et al., described five cases of HSV-1 keratitis in COVID-19 patients that underwent an ophthalmic examination, showing similar symptoms, including photophobia, tearing, decreased vision, eye redness, and pain. After the initial assessment, tests of visual acuity and corneal sensitivity, a fluorescein staining test, and complete anterior and posterior segment examinations were all performed. Researchers confirmed the diagnosis of HSV-1 keratitis in all five cases. Initially, a local and systemic antiviral approach was used in the therapy, in addition to regional antibiotic and mydriatic treatments. The complete reduction of keratitis symptoms and a clear cornea was achieved in all patients within 2 weeks. SARS-CoV-2 infection might constitute a risk factor for developing HSV-1 keratitis, or it might act as a potential activator of this ocular disease [33].

### 4.5. Adie’s Tonic Pupil

Adie’s tonic pupil is a result of damage to the ciliary ganglion or short ciliary nerves and might appear due to an infection, trauma, ischemia, or as a result of surgical procedures [34,35]. Patients are likely to develop progressive miosis, bilateral affection (4% each year), and gradual loss of deep tendon reflexes [36,37]. A case of concurrent tonic pupil and trochlear nerve palsy in a patient with the disease was reported by Ordás et al. A 62-year-old man described his 5-day history of binocular vertical diplopia and blurred vision in the left eye, with an additional dilatation of the left pupil. He suffered flu-like conditions 2 weeks before the disorder. The clinical exam exhibited a right trochlear nerve palsy and a left mydriatic pupil. The MRI, chest X-ray, and analytical results were normal. The antibodies for SARS-CoV-2 were positive (low IgM and high IgG levels). The antiganglioside antibodies were negative. It is presumed that an immune-mediated mechanism was involved in this post-infectious manifestation of COVID-19 [38].

### 4.6. Conjunctival Chemosis

The literature reports describing conjunctival chemosis as a COVID-19 complication are continually increasing. In an Iranian report of 142 hospitalized patients diagnosed with COVID-19, 22 (15.5%) patients presented with chemosis, which is the third-most prevalent symptom after conjunctival hyperemia (28.9%) and tearing (23.2%). A higher frequency of conjunctival chemosis in patients admitted to the ICU compared to those receiving care in other wards was also reported. Furthermore, chemosis is the most common ocular manifestation in ICU-admitted patients. Noteworthy, ocular manifestations are never the first noted symptoms in patients with COVID-19 [39].

Meduri et al., reported that only 1 among 29 hospitalized patients admitted to the COVID-19 center had conjunctival chemosis. Moreover, chemosis coexisted with conjunctivitis with moderate hyperemia and secretions [40].

The researchers from Hubei Province in China documented ocular findings in 12 patients of a group of 38 with confirmed SARS-CoV-2 infection. Amongst the patients with COVID-19-related ophthalmic manifestations, eight patients presented conjunctival chemosis. An interesting correlation between the ophthalmic symptoms and patients with severe systemic manifestations was observed; patients with ocular symptoms were more likely to have more severe systemic manifestations or abnormal findings in blood tests, such as higher white blood cell and neutrophil counts and higher procalcitonin levels, C-reactive protein, and lactate dehydrogenase, compared to patients without ocular symptoms. It was suggested that conjunctival chemosis most commonly occurred in patients with severe systemic disease [26].

Some researchers indicate that the pathogenesis of the conjunctival involvement in COVID-19 patients remains unclear. However, it is assumed that conjunctival involvement might constitute a part of the nonspecific, systemic manifestations rather than infectious conjunctivitis of COVID-19 [41].

## 5. Posterior Segment of the Eye

### 5.1. Central Retinal Vein Occlusion

Central retinal vein occlusion (CRVO) is one of the vascular manifestations associated with the COVID-19 course described in numerous published reports. Sheth et al., described a case of a 52-year-old male with fresh RVO without any comorbidities, such as diabetes, hypertension, or tuberculosis, presenting decreased vision in the left eye since the first day of the disease [42]. Investigations such as fluorescein angiography (FA) and optical coherence tomography (OCT) demonstrated features not different from CRVO in non-COVID-19-related cases. The patient was diagnosed with vasculitic RVO secondary to COVID-19 and treated with oral methylprednisolone and intravitreal injection of ranibizumab. Walinjkar et al., presented a case of a 17-year-old female diagnosed with CRVO secondary to SARS-CoV-2 infection after 21 days from when a cough and fever started [43]. A diagnosis of CRVO was made based on the clinical symptoms, and the blood reports were found to be within the normal limits. A chest CT scan showed a ground glass appearance consistent with COVID-19, but the RT-PCR for SARS-CoV-2 tested negative in her case. The patient’s mother was diagnosed COVID-19-positive with RT-PCR and was discharged from the hospital after testing negative a day before the patient came in with decreased vision in the right eye. A similar case of bilateral central retinal vein occlusion in a 40-year-old patient with COVID-19 pneumonia and right leg DVT has also been described [44]. The patient’s medical history included controlled hypertension and morbid obesity. The clinical diagnosis of bilateral CRVO was based on a clinical fundus examination, OCT, symptoms, and course of the disease.

### 5.2. Central Retinal Artery Occlusion

So far, only four retinal artery occlusions associated with COVID-19 have been reported [45]. One of them was the case of a 60-year-old male with hypertension, dyslipidemia, stable coronary artery disease, and chronic obstructive pulmonary disease, who, on the 12th day of hospital admission due to COVID-19 (confirmed by nasopharyngeal PCR), presented a sudden and painless decrease in vision in his right eye [46]. The diagnosis of central retinal artery occlusion was confirmed during a fundus examination. In the case of a 59-year-old male with hypertension and hyperuricemia, CRAO occurred in the sixth week of hospitalization while the patient was undergoing apixaban anticoagulant treatment [47]. The 48-year-old obese patient was diagnosed with CRAO 2 months after the onset of COVID-19 symptoms [48]. Only one patient, a 54-year-old male, did not have a history of hospitalization or ICU stay and did not have any underlying diseases. The period of onset of COVID-19 until a diagnosis of CRAO was 3 weeks [45]. The authors related the hypercoagulable state of the patient, produced by the inflammation attributable to SARS-CoV-2, with the ophthalmological complication, being consistent with other vascular complications described in the literature, such as ictus or pulmonary embolism [49].

### 5.3. Vitritis and Outer Retinal Abnormalities

Zago Filho et al., reported a case of a 57-year-old female with intraocular inflammation and outer retinal changes developed 12 days after COVID-19 symptoms onset [50]. A color fundus photograph revealed a yellowish lesion within the macular area in both eyes, and OCT demonstrated hyperreflective lesions at the level of the posterior vitreous hyaloid corresponding to the vitritis and at the level of the inner plexiform layer (IPL) and ganglion cell layer (GCL) with disruption of the EZ. The findings remained unchanged for 1 month and were reduced by 2 months in size and reflectivity. Uveitis can be caused by numerous factors, such as herpes simplex virus (HSV), cytomegalovirus (CMV), syphilis, Bartonella, Toxoplasma, Borrelia, and Toxocara, but since none of them was found during the examination, the SARS-CoV-2 infection was presumed to be the cause of the above-mentioned abnormalities.

### 5.4. Acute Retinal Necrosis

The reactivation of acute retinal necrosis in a 32-year-old female after SARS-CoV-2 infection has been reported. The patient, with a prior history of left retinal detachment, which was secondary to necrotizing herpetic retinitis, presented vision loss in the right eye, pain, redness, and photophobia. An ophthalmological examination revealed acute retinal necrosis of the right eye. Additionally, a PCR analysis of the right vitreous was positive for HSV-2, and the RT-PCR was positive for SARS-CoV-2 RNA. Thus, it was suggested that the SARS-CoV-2 infection might cause the reactivation of a latent HSV infection, with its contralateral involvement in patients who have a prior history of HSV-related acute retinal necrosis [51].

### 5.5. Optic Neuritis

Depending on the etiology and clinical manifestations, a visual prognosis and the risk for recurrent injury significantly vary in patients with optic neuritis. A quick and accurate diagnosis may be critical for reducing vision loss and neurologic disability, as well as organ damage [52]. Bennett et al., reported a case of a 44-year-old male with no past medical history that presented 2 weeks after seropositive SARS-CoV-2 infection with vision complications suggesting optic neuritis. Radiological imaging confirmed findings characteristic of acute bilateral optic neuritis. The patient also had his anti-MOG antibodies checked. Whether this was optic neuritis due to SARS-CoV-2, MOG antibody disease, or the activation of MOG antibody disease by COVID-19 has been a topic of discussion and needs further observations in clinical practice [53].

### 5.6. Papillophlebitis

Papillophlebitis is characterized by venous congestion and optic disc edema, which is indicated to appear because of inflammation of the retinal veins or, possibly, the capillaries of the optic disc, leading to venous inefficiency and compression of the central retinal vein [54]. In the reported case of a 40-year-old male, consulted for a slight decrease in sensitivity of the visual field in his left eye (OS), the visual acuities (VA) were 20/20 in both eyes. The OS fundus examination showed dilated and twisted retinal vessels with disc edema and retinal hemorrhages. The patient was diagnosed with papillophlebitis. The OS VA decreased to 20/200 due to macular edema, and he was treated with an intravitreal dexamethasone implant. A performed exploration identified the recent manifestation of and recovery from COVID-19, with inflammation and coagulation alteration. Other systemic diseases were eliminated. A rapid decrease in disc and macular edema after intravitreal dexamethasone injection supported the inflammatory hypothesis. It is assumed that papillophlebitis might occur during COVID-19 disease. It is believed that the inflammatory reaction and the coagulation changes presented could constitute potential risk factors in the development of papillophlebitis [55].

## 6. The Orbital Region

### 6.1. Retro-Orbital Pain

COVID-19 infection might present features that resemble dengue, especially within the first two days after symptom onset. The clinical manifestations may include high-grade fever, headache, retro-orbital pain, muscle and joint pains, and rashes [56,57,58]. However, reports that compare both conditions have demonstrated that retro-orbital pain is more indicative of dengue than COVID-19 infection.

It was reported that a 62-year-old Taiwanese female who had returned to Taiwan from the Philippines presented retro-orbital pain with fever, mimicking Dengue fever. Both Influenza PCR and Dengue virus ELISA had negative results, while the results of the SARS-CoV-2 infection were positive [59]. Joubert et al., reported one similar case while doing a retrospective cohort study to identify predictors of both COVID-19 and Dengue infection. The SARS-CoV-2-positive patient was suffering from retro-orbital pain but tested negative for Dengue [58]. Further, some cases present Dengue–COVID-19 coinfections, in which it is hard to predict which infection indicates which symptoms [60].

### 6.2. Orbital Cellulitis and Sinusitis

Two similar cases described by Turbin et al., presented adolescents with orbital cellulitis and sinusitis with a positive COVID-19 PCR test. Both patients had unusual manifestations, such as intracranial radiographic changes, retro-maxillary antral fat changes, thrombosis, or hemorrhagic abscess. Furthermore, some radiographic findings mimicked a fungal infection, although all fungal tests remained negative. It is unclear whether SARS-CoV-2 infection was a contributing factor associated with the development of symptoms in the described cases or whether this relationship was coincidental. However, a possible explanation is that upper respiratory congestion initiated by SARS-CoV-2 infection could disrupt the mucociliary clearance and induce secondary sinus obstruction, which results in bacterial orbital infection [61].

Shires et al., demonstrated a case of a 76-year-old patient with a history of hypertension, testicular cancer, diabetes, and transient ischemic attack who had a positive SARS-CoV-2 test accompanied by sinusitis, orbital abscess, and osteomyelitis. Furthermore, his tests for MRSA, *Peptoniphilus indolicus*, and *Streptococcus constellatus* were positive. Interestingly, *Peptoniphilus indolicus* is typically found in the vagina or gut biome, not in the eye, which can indicate that the SARS-CoV-2 infection can be related to the presence of bacteria in atypical anatomical regions such as the sinuses or an orbit. Moreover, SARS-CoV-2 infection can also result in avascularity of the nasal cavity and orbit [62].

Another clinical report of a 28-year-old patient with a diagnosis of COVID-19 presented progressive, painful orbital swelling. After imaging tests and clinical examination, clinicians made a diagnosis of SARS-CoV-2-related orbital cellulitis secondary to pansinusitis [63].

A few studies have also reported COVID-19-related orbital cellulitis and sinusitis [55,64,65,66]. It was shown that only 1 among 2742 patients in India presented orbital cellulitis secondary to pansinusitis [66]. Another study reported that 6 (1.4%) out of a group of 425 SARS-CoV-2-positive patients had secondary fungal orbital cellulitis [55]. It was also reported that sinusitis and orbital cellulitis can be associated with post-COVID-19 mucormycosis [65].

## 7. Adnexa of the Eye

### 7.1. Neurogenic Ptosis

SARS-CoV-2 presents neuroinvasive and neurotropic abilities that can result in both central and peripheral nervous system manifestations. The case of a 65-year-old female, who developed new-onset unilateral ptosis and mitosis after being diagnosed with COVID-19, was described by Naor et al. A day after her admission, the patient developed right-sided ptosis and miosis without anhidrosis and was diagnosed with Horner’s syndrome. There were no significant examination, imaging, or laboratory findings, and this may suggest a potential association between the diagnosis of COVID-19 and the development of Horner’s syndrome [67]. In another case of a 10-year-old boy with acute-onset diplopia and ptosis in the right eye, the conducted testing was positive for SARS-CoV-2. The patient was managed successfully with corticosteroids. While isolated post-COVID-19 cranial neuropathies in children are rare and may be encouraged by underlying illnesses, the patient had no comorbidities. Oculomotor nerve palsies caused by inflammation usually present themselves during MRI imaging with findings confined to the oculomotor nerve and without further brain or orbital ones, as seen in said patient. This case led to the heightened suspicion of SARS-CoV-2 infection among children with unusual third nerve palsy [68].

### 7.2. Dacryoadenitis

Infectious dacryoadenitis is predominately caused by viruses, especially the Epstein–Barr virus (EBV); others include adenovirus, mumps, HSV, and HZV type 1 and 2 [69,70]. It was reported that dacryoadenitis might be associated with SARS-CoV-2 infection. In the only reported case, the patient presented common symptoms of dacryoadenitis that included right ocular redness, eyelid swelling, and blurred vision associated with discomfort and pain in the lacrimal gland area. Furthermore, the patient had a history of contact with SARS-CoV-2-infected patients, and his antibody tests for IgM and IgG were positive. However, the patient did not present any typical symptoms of SARS-CoV-2 infection such as cough, congestion, rhinorrhea, fever, dyspnea, diarrhea, malaise, or fatigue. Other tests for autoimmune conditions and infectious diseases, particularly QuantiFERON-TB, HBV and HCV viruses, human immunodeficiency virus (HIV), mumps, adenovirus, EBV, HSV, and Herpes zoster virus (HZV), were all negative. It was then suggested that dacryoadenitis might be a late complication of COVID-19 infection. It is believed that SARS-CoV-2 can penetrate the lacrimal gland via the lacrimal ductiles or a direct hematogenous spread. Another hypothesis assumed that, in the later phase of the COVID-19 infection, an immunological response might cause dacryoadenitis. However, it remains unclear whether dacryoadenitis and the SARS-CoV-2 infection were coincidental or had a causal relationship [56,71].

## 8. Other

### 8.1. Episcleritis

A study from Turkey found a 2.2% prevalence of episcleritis in COVID-19 patients [72]. It was also shown that episcleritis was associated with higher D-dimer levels. In another case, episcleritis developed after the main COVID-19 respiratory symptoms were resolved, and the patient reported to the ophthalmologic clinic with red eyes, foreign body sensations, epiphora, and photophobia. Nodular episcleritis was diagnosed [73]. A case of episcleritis in a 29-year-old male who was diagnosed 3 days before the onset of full-blown COVID-19 has also been reported [74]. About one-third of cases can be associated with viral infections, such as Ebola, hepatitis B virus (HBV), hepatitis C virus (HCV), and herpes zoster virus (HZV), and also, the immune-vascular factors and thrombotic complications of COVID-19 have evoked suspicion in the role of COVID-19 in developing episcleritis [75].

### 8.2. Kawasaki Disease

Children suffer from COVID-19 more mildly, mainly because of more effective immune responses [76]. The prevalence of conjunctivitis in infected children is estimated to be low (1–5%) [77]. However, an increase in morbidity of Kawasaki disease has been noticed, presenting as vasculitis of small and medium vessels that also results in fever, lymphadenopathy, cutaneous and palmar–plantar erythema, conjunctivitis, limb edema, and less frequently, coronary aneurysms [78]. The ocular manifestations are mostly: iridocyclitis, punctate keratitis, vitreous opacities, papilledema, subconjunctival hemorrhage, and conjunctival injection [79]. The majority of patients present with better clinical outcomes in a few days, but some children (0.5–5%) might present toxic shock [78]. A case of a 6-month-old baby with generalized erythema and conjunctivitis has also been described in the literature; the child completely recovered after a treatment with intravenous immunoglobulins (IG) over 48 h [80].

Verdoni et al., conducted a comparative study of two groups—(1) 19 children with a ‘Kawasaki-like’ disease before the start of the SARS-CoV-2 outbreak in Lombardia and (2) 10 children with the disease after the start of the pandemic there [81]. The second group was observed to have had a higher mean age (7.5 years vs. 3 years); the majority had AC antibodies against the virus (8 out of 10) and suffered from a more severe form of the disease: greater cardiac involvement (6 out of 10 vs. 2 out of 19), toxic shock syndrome (5 of 10 vs. 0 of 19), and macrophage activation syndrome (5 of 10 vs. 0 of 19). Thus, SARS-CoV-2 could have been a strong stimulus in the host capable of triggering a disproportionate immune response. The predictors of this response are still unknown.

### 8.3. Opthalmoparesis Consistent with Abducens Nerve Palsies

Two cases of patients with a diagnosis of COVID-19 after presenting with diplopia and ophthalmoparesis were described [82]. The combination of ophthalmoparesis with bilateral abducens nerve palsies, leg paresthesia, and areflexia in the first case could suggest acute demyelinating inflammatory polyneuropathy secondary to a virus-mediated immune response. In the second case, although the radiological evidence of abducens nerve involvement was missing, the presence of painless diplopia and abduction palsy of the right eye might reflect viral leptomeningeal invasion. The event of cranial neuropathies ought to provoke the consideration of SARS-CoV-2 infection in patients with even gentle symptoms and signs of SARS-CoV-2 infection.

### 8.4. Oculomotor Nerve Palsy

The clinical presentation of oculomotor nerve palsy includes ptosis and the restriction of adduction, elevation, and depression movements of the eyeball. The case of a 55-year-old male with confirmed SARS-CoV-2 infection and diagnosed with third cranial nerve palsy was described. The patient was treated supportively for his infection and remained stable on room air during his hospitalization. No connective factors other than COVID-19 were identified as a cause for his cranial third nerve palsy, which resolved spontaneously during outpatient follow-up. The pathogenesis and prognosis of cranial nerve palsy in SARS-CoV-2 infection are still unclear [83]. Another reported case of acquired non-pupil-sparing oculomotor nerve palsy in a previously asymptomatic child indicated a possible link to SARS-CoV-2 infection. The patient, in contrast to oculomotor nerve palsies caused by presumed inflammation, presented no findings in the MRI. Regarding the patient’s spontaneous improvement, researchers hypothesized that the damage to the oculomotor nerve caused by SARS-CoV-2 was not permanent and that oculomotor nerve palsy could resolve spontaneously within a short time [84].

### 8.5. Cerebrovascular Accident (CVA) with Vision Loss

Vision loss can be the most disabling residual effect after cerebral infarction; temporary vision problems can equally be an indication of stroke, and a prompt evaluation after recognition of the visual signs can prevent future vascular injury [85]. In the COVID-19 era, vigilance for cerebrovascular complications of the aforementioned illness is needed. Scientists presented a case of bilateral occipitotemporal infarction observed as a sudden cortical loss of vision with hemorrhagic transformation after intravenous thrombolysis in a patient with diabetes infected by SARS-CoV-2 [86]. It is currently known that SARS-CoV-2 can penetrate the brain, leading to neuronal defects. Moreover, it is suggested that SARS-CoV-2 could, due to its cerebrovascular system effects, possibly provoke neuronal complications. Early data show that stroke can be one of the leading neurological complications in COVID-19 patients [87].

### 8.6. Acute Angle Closure Glaucoma and Horner’s Syndrome as Complications of ICU Treatment

Nerlikar et al., reported the case of a 53-year-old male with ocular discomfort and blurred vision in both eyes that was caused by prone positioning during ventilation for COVID-19 pneumonia. The patient was under sedation and was placed for 8 h in a prone position every day for 2 weeks. Additionally, he could not manifest his symptoms, so the detection and treatment were shifted over time. Moreover, it was also suggested that the medication that was given to the patient, such as Glycopyrrolate, Noradrenaline, and Salbutamol, could cause acute angle closure. Another possible reason for elevated intraocular pressure could be steroids [88].

A similar study described a patient admitted to the ICU due to COVID-19 who developed acute closure glaucoma after the use of ipratropium bromide and salbutamol, darkroom conditions, and prone positioning for 3 weeks. Despite treatment with eye drops and cataract surgery, the patient lost vision in her right eye [89]. Other studies have also indicated that some anticholinergics, sympathomimetics, and other drugs may induce AACG [90,91].

Other researchers strive to explain the main mechanisms of acute angle closure glaucoma caused by drugs. The first hypothesis assumes pupillary block and iridocorneal angle closure, and the second one blames the mass effect, which causes anterior displacement of the lens–iris diaphragm [92]. According to some studies, SARS-CoV-2 infection is not directly related to acute angle-closure glaucoma; however, it possibly contributes to AACG due to a prolonged prone position and some medications used in the treatment of COVID-19-related pneumonia.

Horner’s syndrome is characterized by ptosis, miosis, enophthalmos, and, rarely, a lack of sweating on the affected side of the face [93]. So far, the data on COVID-19-related Horner’s syndrome are still limited, but some reports have confirmed a correlation. The first report presented a 65-year-old female who tested positive for SARS-CoV-2 and was admitted to the hospital due to hypoxemia. The next day, she developed ptosis and miosis and was diagnosed with Horner’s syndrome. Despite the lack of an unequivocal mechanism and significant examination explaining the appearance of Horner’s syndrome, in this case, the influence of the SARS-CoV-2 infection could be associated with the development of ptosis and miosis 2 days after the COVID-19 diagnosis [67].

Other researchers described a 38-year-old patient with left ptosis, fever, general weakness, mild headache, slight left pupil construction, and left enophthalmos. Horner’s syndrome was diagnosed based on a neurological examination. Further, the chest CT and RT-PCR test detected SARS-CoV-2 infection. The mechanism of this syndrome, in this case, is debatable. It was suggested that it could be related to the inflammation of the upper lobe of the lungs or a reactive enlargement of the cervical lymph nodes. Another reason might be the direct impact of the virus on the nervous system [94].

### 8.7. Miller Fisher Syndrome

There have been several cases describing post-COVID-19 Miller Fisher syndrome, and they all presented similar symptoms and evolution [95,96,97,98,99,100,101]. Neurological symptoms were usually observed 5–20 days after the COVID-19 diagnosis [95,96,97,98,99,100,101]. The most common symptoms related to MFS included paresthesia, ophthalmoplegia, blurred vision, ataxia, areflexia, and others. It remains uncertain whether post-COVID-19 MFS is induced by viral neurotropism or the disturbed immune response. The presence of a GQ1b antibody, increased proinflammatory cytokines in the plasma, and the absence of SARS-CoV-2 in the cerebrospinal fluid suggest immune-mediated injury. However, when the testing for anti-GQ1b is negative, the pathogenesis could be explained by the neuroinvasive capacity of SARS-CoV-2 [95,102]. Generally, most cases of MFS show positive anti-GQ1b, but it is not an obligatory sign of a MFS diagnosis. Moreover, the presence of GQ1b antibodies indicates a faster recovery. According to the studies, most patients have responded well to intravenous immunoglobulin [95]. Of note, post-COVID-19 MFS was also reported in a 7-year-old child [98].

### 8.8. Xerophtalmia

A vitamin A deficiency might lead to various ocular symptoms, including conjunctival and corneal xerosis, Bitot’s spots, keratomalacia, nyctalopia, and retinopathy, which refer to xerophthalmia. So far, the data on xerophthalmia as a COVID-19 complication are still limited [103,104,105]. The research paper about ocular manifestations and clinical characteristics of 535 cases of COVID-19 in Wuhan, China, reported that some patients with COVID-19 had chronic ocular diseases, such as xerophthalmia (24 patients, 4.5%). However, this conclusion was found additionally and was not the subject of the study. The association between COVID-19 and xerophthalmia still remains unclear [21]. Other researchers referenced this study showing that, so far, no other study has been performed on this matter, and the knowledge about xerophthalmia as a complication of SARS-CoV-2 infection is still insufficient [106,107].

### 8.9. Mucormycosis

SARS-CoV-2 infection might induce opportunistic fungal infections, such as pulmonary aspergillosis, pneumocystis pneumonia, oral candidiasis, or, rarely, mucormycosis [108]. Despite that, the amount of the literature reporting COVID-19-related mucormycosis is continually increasing. A major risk factor is the use of corticosteroids while managing a severe course of COVID-19. Additionally, it was reported that diabetic patients are more predisposed toward acquiring mucormycosis [109,110,111,112]. Other risk factors include neutropenia and hematologic cancers, stem cell transplant patients, and immunity-reduced patients [113]. The most common predilections were nasal, rhino-orbital, and rhino-orbital–cerebral mucormycosis [110,114,115].

A recent review by Singh et al., showed that SARS-CoV-2-infected males (78,9%) were more prone to mucormycosis than females. Furthermore, 80% of the patients had diabetes and 14.9% presented diabetic ketoacidosis. Corticosteroids were used in 76.3% of the cases. The most common was mucormycosis of the nose and sinuses, and the second was rhino-orbital. Fatalities were reported in 30% of the cases [114].

### 8.10. Endogenous Endophthalmitis

Endogenous endophthalmitis is usually reported in patients with numerous coexisting comorbidities, as well as those with prolonged hospitalization, intravenous mediations, or corticosteroid/immunosuppression therapy. Nayak et al. (2021), in their cohort study, presented that SARS-CoV-2 infection might be associated with an onset of endogenous endophthalmitis. The researchers observed that the prolonged administration of three drugs—namely, systemic corticosteroids, broad-spectrum antibiotics, and IL-6 inhibitors (tocilizumab)—predisposed the patients toward the onset of endogenous endophthalmitis [116]. Additionally, Bilgic et al. (2021) reported three cases of endogenous bacterial endophthalmitis in patients during their COVID-19 recovery stage [117].

## 9. Ophthalmic Manifestation in Children

Children tend to present a milder course of COVID-19 and significant lower mortality rates compared to adults. Data regarding an ophthalmic manifestation in children are still limited; however, there have been some reports describing COVID-19-associated ophthalmic manifestations in children.

A cross-sectional study from China indicated that almost one-quarter of hospitalized children with COVID-19 present with ocular manifestations. The most common included conjunctival discharge, eye rubbing, and conjunctival congestion. Noteworthy, the development of ocular symptoms was more likely to appear in children with systemic symptoms such as a fever or cough. Moreover, the ophthalmic manifestations were milder, and children recovered from it very quickly without long-term complications [118].

Researchers from Spain also reported a few cases of post-COVID-19 ocular manifestations in children. Six out of seventeen children presented ophthalmic symptoms such as conjunctivitis, episcleritis, retinal vasculitis, and retrobulbar optic neuritis. Of note, only the SARS-CoV-2 IgG antibodies were positive, while the COVID-19 PCR tests were negative, which led to the conclusion that ocular manifestations might be a complication of past COVID-19 [119].

Wu et al., presented a COVID-19-positive child 2 years and 10 months old who only revealed ophthalmic symptoms such as conjunctivitis and eyelid dermatitis without any systematic manifestations. After treatment, the symptoms gradually disappeared after 5 days [80]. Eleiwa et al., reported a case of a SARS-CoV-2-positive 10-year-old patient with lateral rectus myositis, enlarged lacrimal gland, and signs of orbital inflammation. However, it remains unclear whether the SARS-CoV-2 infection had an influence on the orbital inflammation or if it was coincidental [120].

On the other hand, Pérez-Chimal et al., reported ophthalmic manifestations associated with SARS-CoV-2 in newborn infants. All 15 newborns presented edema and hyaline secretion, 11 of them had chemosis and hemorrhagic conjunctivitis, and 8 had retinal findings. One infant presented with rubeosis and posterior synechiae. The detailed mechanism is unknown, but a possible explanation could be associated with prematurity, mechanical ventilation, hemodynamic compromise, or SARS-CoV-2 infection as well [121] (Table 2).

## 10. Conclusions

In this article, several types of ophthalmic manifestations in COVID-19 patients were discussed. The first group involves inflammations: mild, bilateral conjunctivitis, mainly without the corneal affectations, episcleritis, viral keratoconjunctivitis, hemorrhagic and pseudomembranous conjunctivitis, viritis and outer retinal abnormalities, papillophlebitis, dacryoadenitis (acute or chronic inflammation of the lacrimal gland), keratitis, acute retinal necrosis, optic neuritis, conjunctival chemosis, orbital cellulitis, and sinusitis. The next group includes vascular impediments: central retinal vein or artery occlusion and Kawasaki disease. The following group includes neurological complications: ophthalmoparesis consistent with abducens nerve palsies, oculomotor nerve palsy, neurogenic ptosis, Adie’s tonic pupil, Miller Fisher syndrome. Furthermore, we acknowledged acute angle closure glaucoma and Horner’s syndrome as complications of ICU treatment and vitamin A deficiency—xerophthalmia. SARS-CoV-2 infection might also lead to opportunistic fungal infections; among which, mucormycosis seems to be rarely distinguished.

It is immensely important to recognize and gain knowledge concerned with the ophthalmic manifestations of COVID-19 disease, with the aim of accurate treatment introduction and the prevention of the unwanted consequences of the infection, improving the quality of life of infected patients and, at the same time, decreasing the number, as well as the severity, of possible side effects due to infection.

## Figures and Tables

**Table 1:** Comparison of typical and atypical COVID-19 infection symptoms.

Typical Manifestations	Atypical Manifestations
Fever	Kawasaki-like disease
Cough	Cerebrovascular disorders
Fatigue	Cutaneous manifestations
Headache	AKI
Loss of smell and taste	Conjunctival congestions
Dyspnea	Seizure

**Table 2:** Summary of case reports presented in the review.

Ref.	Author	Anatomical Structure Affected	Type of COVID Test	Outcome of the Case	Case Details
[18]	Alnajjar et al.	conjunctiva and cornea; later, lungs	RT-PCR	It is important to increase the awareness of atypical presentation for COVID-19 infection.	A case that was initially presented with keratoconjunctivitis with the appearance of respiratory symptoms four days later. The improvement was showed within four days of successful treatment for both COVID pneumonia and ocular disease.
[29]	Scalinci et al.	conjunctiva	RT-PCR	Authors emphasize the importance of eye protection, even if patients do not show typical signs of infection.	In these patients, acute conjunctivitis was the presenting sign and symptom, but also remained the sole form of manifestation of COVID-19.
[122]	Salducci et al.	conjunctiva, lungs	Oropharyngeal and nasal swab RT-PCR	COVID-19 may be detected in the tears and conjunctival secretions in novel coronavirus pneumonia patients with conjunctivitis, more research needs to be carried out in order to confirm its ability to infect ocular tissue.	Severe viral conjunctivitis in a patient diagnosed with COVID-19, characterized by both red, irritated and swollen eyes, with transparent serous secretions, conjunctival chemosis, pseudomembranes of fibrin, and inflammatory cells on the tarsal conjunctiva.
[74]	Otaif et al.	episclera	RT-PCR	Episcleritis can be a possible presenting sign of COVID-19. Understanding the association between ocular signs/symptoms and COVID-19 can aid in the diagnosis of the viral infection and can help in limiting its transmission.	A 29-year-old man with no prior medical condition presented with a complaint of redness and foreign body sensation in his left eye. He had no symptoms in his fellow eye, nor did he have any systemic symptoms.
[75]	Mangana et al.	episclera	RT-PCR	This case illustrates episcleritis as a possible ocular complication of COVID-19.	A 31-year-old woman presented cough and myalgia without fever. On the next day, symptoms disappeared but were followed by anosmia and ageusia. After they resolved, no other general signs or symptoms appeared except the ocular symptoms (red eye, foreign-body sensation, epiphora and photophobia without impaired visual acuity).
[31]	Guo et al.	conjunctiva	RT-PCR	COVID-19 can be complicated by relapsing viral keratoconjunctivitis and the topical cytokine involvement surge in the pathogenesis of COVID-19 as it relates to viral keratoconjunctivitis.	A 53-year-old man confirmed with COVID-19 developed symptoms of viral conjunctivitis in the left eye approximately 10 days after the onset of COVID-19. The patient was subsequently diagnosed with viral keratoconjunctivitis in both eyes 5 days after the symptoms in the left eye were satisfactorily relieved. The disease progressed rapidly, with spot staining observed at the periphery of the corneal epithelium.
[32]	Navel et al.	conjunctiva, lungs	Analyses of bronchial secretions with RT-PCR	External ocular infections could be factors of infectious spreading. Physicians should be aware of late ocular complications in COVID-19 patients to prevent sequelae.	A 63-year-old male was admitted in intensive care unit, seven days after the beginning of an influenza-like symptoms, to manage an acute respiratory syndrome related with SARS-CoV-2. At day 19, ocular examination described petechias and tarsal hemorrhages, mucous filaments and tarsal pseudomembranous.
[42]	Sheth et al.	retinal vein	RT-PCR of sputum samples	This case supports the mechanism of thrombo-inflammatory state secondary to the “cytokine-storm” as the pathogenesis for systemic manifestations of COVID-19.	A unique case of vasculitic retinal vein occlusion secondary to COVID-19 in a 52-year-old patient who presented with the diminution of vision in the left eye 10 days after he tested positive for SARS-CoV-2. All investigations for vasculitis were negative.
[43]	Walinjkar et al.	central retinal vein, lungs	COVID-19 Immunoglobulin test	A case of central retinal vein occlusion (CRVO) in a patient with proven past history of COVID-19, indicates the disease as the possible etiology.	A 17-year-old female who presented with central retinal vein occlusion and had a proven recent past COVID-19 infection.
[44]	Gaba et al.	central retinal vein, lungs, deep femoral vein	RT-PCR	High index of suspicion for retinal vein occlusion should appear in case of patients with COVID-19 infection who present blurred vision and severe pneumonia.	A 40-year-old man who presented with a 3-day history of shortness of breath, cough, and fever. He also reported right calf pain and blurring of vision in both eyes. His medical history included hypertension and morbid obesity. The patient was found to have severe COVID-19 pneumonia on high-resolution computed tomography of the chest, right leg deep venous thrombosis on Doppler ultrasonography, and bilateral central retinal vein occlusion (RVO) on fundal examination.
[45]	Ucar et al.	macula, retina, central retinal artery	RT-PCR	Central retinal artery occlusion is a possible result of the COVID-19 infection.	A 54-year-old male patient applied with a sudden and painless loss of vision in his right eye. He was suffering from COVID-19. His best-corrected visual acuity of the right eye was finger counting from 30 cm. The fundus examination revealed the presence of a ‘cherry-red spot’ appearance in the right eye. In optical coherence tomography imaging, hyper-reflectivity was observed in the inner retinal layers as well as increased retinal thickness in the right eye. In fundus fluorescein angiography, delayed arterial filling and prolonged arteriovenous transit time were observed in the right eye.
[46]	Acharya et al.	central retinal artery, lungs	RT-PCR	Hypercoagulability associated with COVID-19 has been described as a “sepsis-induced coagulopathy” and may predispose to spectrum of thromboembolic events.	A 60-year-old male with past medical history of hypertension, dyslipidemia, stable coronary artery disease and chronic obstructive pulmonary disease presented with persistent fever, cough, and worsening shortness of breath. Revealed bilateral reticular interstitial opacities consistent with viral pneumonia. Subsequently he developed acute respiratory distress syndrome with cytokine release. On the twelfth hospital day, he complained of sudden onset of painless loss of vision in the right eye. Upon examination the right pupil was unresponsive to light and absent accommodation reflex. During the posterior segment examination, it was found that the right optic nerve had slightly indistinct margins and a cherry red spot with significant retinal whitening.
[47]	Dumitrascu et al.	ophthalmic artery, lungs	RNA Reverse Transcriptase-PCR	Ocular vascular complications may be reported in COVID-19 patients.	A 48-year-old man with obesity was hospitalized with a severe form of COVID-19 infection, complicated with acute respiratory failure, septic shock, dilated cardiomyopathy and fungemia. Patient developed acute severe right eye visual loss of no light perception and was diagnosed with incomplete ophthalmic artery occlusion. Stroke etiological work-up found no embolic sources, resolution of the dilated cardiomyopathy and negative antiphospholipid antibodies.
[48]	Montesel et al.	central retinal artery	RT-PCR	It is likely that COVID-19 patients could be at risk of developing retinal vascular occlusions. A focused ophthalmological surveillance is advisable to prevent and manage this possible cause of severe vision loss that has an important impact in health care system.	The patient was a 59-year-old male, African ethnicity, with a previous longstanding history of hypertension and hyperuricemia under treatment (Table 1). He was hospitalized four days after the onset of fever, dry cough and progressive dyspnea. His state deteriorated and the ICU stay was complicated by a bacterial pneumonia and a stage 3 acute renal injury that required hemodialysis. The patient remained intubated for a total of 10 days, five of which with prone positioning. Cerebral ischemic events were diagnosed with computed tomography angiography and magnetic resonance imaging. One week after discharge from the hospital, he presented to our emergency service complaining of painless vision loss in the left eye.
[50]	Zago Filho et al.	vitrea, retina	IgM and IgG serological tests	Not only the inner retinal layers, but also vitreal and outer retinal layer illness might be caused by COVID-19.	A 57-year-old woman was seen 12 days after COVID-19 symptoms onset. Spectral-domain optical coherence tomography demonstrated hyperreflective pinpoints at the level of posterior vitreous hyaloid, corresponding to vitritis, hyperreflective lesions at the level of inner plexiform and ganglion cell layers, and disruption of the ellipsoid zone.
[80]	Wu et al.	Eyelid, conjunctiva,	RT-PCR, serological tests	Doctors should not forget to conduct COVID-19 screening when children come to hospital for ocular abnormalities.	The child, 2 years and 10 months old, on day 7 of confinement, the child presented with conjunctivitis and eyelid dermatitis. CT revealed myocardial damage and atypical change in lymphocyte count. After treatment conjunctivitis and eyelid dermatitis gradually disappeared 5 days later.
[51]	Gonzalez et al.	retina	RT-PCR	Case suggests that COVID-19 may cause a latent HSV infection to reactivate, causing contralateral involvement in patients with a prior history of HSV-associated acute retinal necrosis.	A 32-year-old female with a distant history of left retinal detachment secondary to necrotizing herpetic retinitis complained of right-eye vision loss, pain, redness, and photophobia. An ophthalmological examination revealed findings consistent with acute retinal necrosis of the right eye. A polymerase chain reaction (analysis of the right vitreous was positive for herpes simplex virus type 2. A coronavirus disease 2019 (COVID-19) screening test was positive.
[123]	Haider et al.	Posterior Communicating Artery, Oculomotor Nerve	None	Early recognition and evaluation of palsy of the third cranial nerve is important in order to rule out a potential PCOM aneurysm.	The patient presented with image verified aneurysm in addition to symptomatology congruent with this finding, but upon intraoperative evaluation, was found to have oculomotor nerve compression more likely attributable to a tortuous PCOM. Upon clipping of the aneurysm and placement of a felt pledget between the aberrant artery and nerve in question, amelioration of symptoms was appreciated.
[83]	Douedi et al.	oculomotor nerve	RT-PCR	The pathogenesis and prognosis of cranial nerve palsy in COVID-19 patients is still unclear. This case emphasizes the need for continued symptom monitoring and identification in patients diagnosed with COVID-19.	A case of a 55-year-old male with confirmed COVID-19 infection presenting with third cranial nerve palsy. Since his hospital course remained unremarkable, he was treated supportively for his COVID-19 infection and remained stable on room air during his hospitalization. No causative factors other than COVID-19 were identified.
[84]	de Oliveira et al.	Oculomotor nerve	serological tests	Oculomotor nerve palsy may appear among asymptomatic COVID-19 children.	The case of a 2-year-old girl with acute-onset divergent strabismus and ptosis in the right eye. She had an exotropia of 45^Δ^ for near, eyelid ptosis affecting the visual axis, adduction, limitations of up- and downgaze, and a discrete mydriasis in the right eye.
[67]	Naor et al.	Oculomotor nerve	RT-PCR	Horner’s syndrome can be one of the symptoms of COVID-19.	A 38-year-old, right-handed male patient was referred to the Emergency Department due to ptosis, general weakness, fever, and mild headache.
[68]	Elenga et al.	Oculomotor nerve	RT-PCR	There should be a heightened suspicion of occult COVID-19 infection among children presenting with unusual III nerve palsy.	The case of a 10-year-old boy with acute-onset diplopia and ptosis in the right eye.
[53]	Sawalha et al.	Optic nerve	serological tests	Whether this was an optic neuritis due to COVID-19, MOG antibody disease, or an activation of MOG antibody disease by COVID-19 is discussed in this case.	A 44-year-old male patient with no past medical history presented 2 weeks after seropositive COVID-19 infection with vision problems suggestive of optic neuritis. Radiological testing showed findings suspicious for acute bilateral optic neuritis. The patient had also anti-MOG antibodies.
[54]	Insausti-García et al.	Retina, central retinal vein, optic disc	RT-PCR	SARS-CoV2 may have acted as a risk factor for the development of papillophlebitis.	A 40-year-old white male with the main complaint of persistent and painless decrease in the sensitivity of his vision in his left eye. The patient indicated that 6 weeks before the onset of visual symptoms, he had presented high fever, persistent cough, and myalgia for approximately 2 weeks.
[35]	Batawi et al.	Pupil	None	Dilute pilocarpine 0.1% three times a day can be considered as a pharmacological therapy for symptomatic relief of Adie’s tonic pupil.	A 40-year-old healthy man presented with a 4-month history of photophobia, blurred vision and a right dilated pupil. Examination revealed a right pupil that was not reactive to light but constricted strongly to a near target and slowly redilated when he looked back in the distance.
[38]	Ordás et al.	Pupil, trochlear nerve palsy	serological tests	Adie’s pupil can be a postinfectious manifestation of COVID-19.	A 62-year-old man reported a 5-day history of binocular vertical diplopia and blurred vision in his left eye, noticing that his left pupil was dilated. He had suffered a flu-like syndrome 2 weeks before. Clinical exam showed a right trochlear nerve palsy and a left mydriatic pupil. MRI, X chest ray, and analytical results were normal.
[33]	Majtanova et al.	cornea	RT-PCR	SARS-CoV-2 infection may be a risk factor for developing HSV-1 keratitis, or it may act as a potential activator of this ocular disease.	In total, five COVID-19 patients underwent ophthalmic examination, showing similar symptoms, including photophobia, tearing, decreased vision, eye redness, and pain. After initial assessment, tests of visual acuity and corneal sensitivity, a fluorescein staining test, and complete anterior and posterior segment examinations were performed. A diagnosis of HSV-1 keratitis was confirmed in all cases.
[86]	Bonardel et al.	posterior cerebral artery	RT-PCR	In time of SARS-CoV2 pandemic, neurologists need to be vigilant for cerebrovascular complications of COVID-19.	A case of bilateral occipitotemporal infarction revealed by a sudden cortical blindness with hemorrhagic transformation after intravenous thrombolysis in a diabetic patient infected by COVID-19.
[71]	Díaz et al.	Lid, lacrimal gland	RT-PCR	Orbital inflammatory disease due to infectious process or immunological response may potentially occur in COVID-19 patients.	A 22-year-old previously fit and healthy male presented with 4-day history of right ocular redness, eyelid swelling, and blurred vision associated with discomfort and pain in the lacrimal gland area. He was found to have right acute dacryoadenitis based on clinical examination and orbital imaging. One day after initiation of oral antibiotic and non-steroidal anti-inflammatory therapy, he developed worsening of the orbital inflammation and partial ophthalmoplegia. The patient did not have any systemic features of COVID-19, but he was in close contact with his mother and with his partner who both had respiratory symptoms and tested positive.
[89]	Lozano et al.	iridocorneal angle	ND	Patients who require sedation, mechanical ventilation, a prolonged stay in the ICU, and multiple medications are at higher risk of developing serious ocular complications; thus, the ICU is an unsafe environment for the eye.	A 60-year-old female complained of photophobia, pain, and vision loss in her right eye while hospitalized in the ICU for COVID-19 management. Symptoms developed. Despite management with hypotensive eye drops and cataract surgery, the patient developed bilateral glaucomatous damage and vision loss in her right eye.
[88]	Nerlikar et al.	iridocorneal angle	ND	With prone position ventilation being a commonly used adjuvant treatment for acute respiratory distress syndrome associated with COVID-19 pneumonia, acute angle closure may be precipitated in these patients if they have pre-existing narrow angles.	A case of bilateral acute angle closure glaucoma developing after prone position ventilation for severe COVID-19 pneumonia.
[94]	Popiołek et al.	Oculomotor nerve	RT-PCR	A COVID-19 pneumonia can be complicated by Horner’s Syndrome.	A 38-year-old, right-handed male patient was referred due to ptosis, general weakness, fever, and mild headache. He did not complain of dyspnea, cough, or any other upper-respiratory tract symptoms. Findings from the neurologic exam, at admission, included: left ptosis, slight left pupil constriction in the dim light, and left enophthalmos.
[57]	Ruiy et al.	retro-orbital space	RT-PCR	A COVID-19 infection may mimic dengue fever.	A reported case of COVID-19 with fever, headache and retro-orbital pain after a week-long trip to the tropics or subtropics, which mimics the classic manifestation of dengue fever.
[59]	Saipen et al.	retro-orbital space, muscles, liver, joints, skin	RT-PCR	It is important to consider the possibility of COVID-19 in patients positive for dengue and vice versa, since the result will affect management and prognosis.	A female aged 62 years with hypertension presented with body malaise and fever. Two days before her admission, the patient started to experience high-grade fever with associated headache (frontoparietal in location, rated 5/10 and band-like in character) and retro-orbital pain, generalized body ache, myalgia, and arthralgia.
[60]	Verduyn et al.	muscles, joints, skin	RT-PCR	In tropical areas where arboviruses and COVID-19 may coexist, clinical diagnosis is difficult, and patients should be tested for both viruses.	A case of an 18-year-old male, with no relevant past medical history except occasional migraines. The onset of symptoms occurred with fever (39 °C), asthenia, anorexia, and headache. He tested positive in the emergency department for SARS-CoV-2.
[98]	Biswas et al.	Autonomic nerves	ND	Post-SARS-CoV-2 infection state may induce neurological manifestations as well as autonomic dysfunction, that preceded the fully developed clinical triad of Miller Fisher syndrome.	A unique case of a patient infected with SARS-CoV-2 who acutely presented with autonomic dysfunction. She was finally diagnosed to be a case of anti-ganglioside antibody positive post-COVID-19 MFS with dysautonomia and treated with intravenous immunoglobulin with an excellent response.
[99]	Reyes-Bueno et al.	peripheral nervous system, central nervous system	RT-PCR, serological tests	Miller Fisher syndrome can be associated with SARS-CoV-2 infection.	A 51-year-old female diagnosed with Miller Fisher syndrome two weeks after COVID-19. RT-PCR to SARS-CoV-2 was negative but IgG was positive.
[100]	Manganotti et al.	central nervous system, lungs	ND	In conclusion, this case report describes the characteristics of an MFS/cranial polyneuritis in a patient with COVID-19, and the clinical responses to intravenous immunoglobulin therapy.	A 50-year-old woman that developed SARS-CoV-2 pneumonia and was admitted at the COVID-19 dedicated unit where she developed neurological symptoms 10 days after admission. After neurological examination, a diagnosis of Miller Fisher syndrome was hypothesized
[102]	Lowery et al.	Lungs, peripheral nervous system, central nervous system	RT-PCR	The case demonstrates the severe neurological implications, prolonged recovery and implications in the concomitant respiratory failure of COVID-19 patients with neurological symptoms on the spectrum of disorders of Guillain Barre Syndrome.	The clinical course of a 45-year-old immunosuppressed man is summarized as a patient who developed ataxia, ophthalmoplegia, and areflexia after upper respiratory infection symptoms began.
[61]	Turbin et al.	Epidura, meningea, maxillary antrum fat	RT-PCR	These cases highlight two unusual orbital presentations of cellulitis occurring in the context of SARS-CoV-2 co-infection.	We reviewed two cases of adolescents with orbital cellulitis, sinusitis, and SARS-CoV-2 infection. Unusual clinical and radiographic characteristics included hemorrhagic abscess with blood of varying age in the first, intracranial epidural abscess in the second, radiographic signal consistent with hemorrhagic or thrombotic phenomena, retro-maxillary antral fat changes, and meningeal enhancement or extension in both cases.
[62]	Shires et al.	Periorbital space, sinus	RT-PCR	Given the concomitant infection with COVID-19 and unusual presentation, the patient’s sinus cultures support the notion that COVID-19 can affect the presence of bacteria within certain anatomical regions.	The patient is a 76-year-old male and was sent from a nursing facility for left eye drainage and psychiatric evaluation. Upon presentation, the patient was not fully oriented and could not provide a history of the eye drainage. CT scan showed sinusitis with left orbital and periorbital abscess formation, cellulitis, and extensive osteomyelitis.
[110]	Garg et al.	Lungs	RT-PCR	Physicians caring for critically ill COVID-19 patients must be aware of serious infections that can complicate the course of COVID-19. A high degree of clinical suspicion is required to diagnose pulmonary mucormycosis. Early diagnosis and timely management are necessary to improve outcomes in pulmonary mucormycosis.	A 55-year-old man with long-standing diabetes mellitus, hypertension, and ischemic cardiomyopathy presented with fever, dry cough, and progressive breathlessness of three days duration. He was diagnosed with type 2 diabetes mellitus ten years before the current illness.
[113]	Werthman-Ehrenreich et al.	Orbital compartment, ethmoid sinus, lungs, central nervous system	ND	It is impossible to know for certain whether this patient’s COVID-19 infection was contributory to her illness or merely coincidental.	A 33-year-old Somali female with past medical history of hypertension and asthma, presented to the emergency department with altered mental status. Upon examination, she appeared in moderate distress with acutely altered mental status. Most notable was left eye ptosis with 1 cm proptosis. The eye had a fixed dilated pupil with complete ophthalmoplegia.
[124]	Maini et al.	periorbital space, central nervous system, paranasal sinuses	RT-PCR	Research needs to be carried out in COVID-19 patients for better prevention and management of opportunistic infections in order to reduce its incidence and morbidity.	A case of post COVID-19 Sino-orbital Mucormycosis infection caused by Rhizopus oryzae and its management.

Abbreviations: ND—no data.

## Data Availability

Not applicable.

## Abbreviations

AACG	acute angle closure glaucoma
ACE2	angiotensin-converting enzyme 2
AKI	acute kidney injury
ARDS	acute respiratory distress syndrome
ARN	acute retinal necrosis
CMV	cytomegalovirus
COVID-19	coronavirus disease 2019
CT	computed tomography
EBV	Epstein-Barr virus
ECs	epithelial cells
HBV	hepatitis B virus
HCV	hepatitis C virus
HIV	human immunodeficiency virus
HSV	herpes simplex virus
HZV	Herpes zoster virus
ICU	intensive care unit
MFS	Miller Fisher syndrome
OCT	optical coherence tomography
SARS-CoV-2	severe acute respiratory syndrome coronavirus 2
VZV	varicella-zoster virus
